# Increased neutrophil senescence is associated with impaired immunosuppressive activity in systemic lupus erythematosus: Neutrophil senescence and immunosuppression in SLE

**DOI:** 10.3724/abbs.2025047

**Published:** 2025-06-24

**Authors:** Lei Han, Fengling Huang, Qingchen Zhu, Huan Wang, Tianlin Lu, Chunyuan Xiao, Jing Xu, Xiaoyan Zhang, Yichuan Xiao, Xinfang Huang

**Affiliations:** 1 Department of Rheumatology Shanghai East Hospital Tongji University School of Medicine Shanghai 200120 China; 2 CAS Key Laboratory of Tissue Microenvironment and Tumor Shanghai Institute of Nutrition and Health University of Chinese Academy of Sciences Chinese Academy of Sciences Shanghai 200031 China; 3 Department of Pharmacy Hebei Medical University Shijiazhuang 050011 China; 4 Department of Renal and Rheumatology Xin Hua Hospital Affiliated with Shanghai Jiao Tong University School of Medicine Shanghai 200092 China

**Keywords:** systemic lupus erythematosus, neutrophils, senescence, inhibitory capacity, reactive oxygen species

## Abstract

Systemic lupus erythematosus (SLE) is an autoimmune disease characterized by a complex pathogenesis that was previously thought to involve primarily adaptive immunity. Emerging evidence underscores the role of neutrophils in shaping immune dysregulation and inducing organ damage in lupus. This study aims to investigate the dynamics of neutrophil senescence and its relationship with lupus, an area that remains poorly understood. Here, we identify a significantly elevated proportion of CXCR4 ^hi^CD62L ^lo^ senescence-like neutrophils in the peripheral blood of SLE patients compare to that in the healthy donors. Increased numbers of senescence-like neutrophils are positively correlated with SLE disease activity and autoantibody production in SLE patients. In addition, senescence-like neutrophils derived from SLE patients exhibit an impaired ability to suppress the proinflammatory activity of natural killer (NK) cells and CD4 ^+^ T cells. Further mechanistic exploration suggests that these senescence-like neutrophils might exert their immunosuppressive effects via reactive oxygen species (ROS) production under physiological conditions. Our results demonstrate that senescence-like neutrophils could serve as biomarkers for assessing the disease activity of SLE. The compromised immunosuppressive function of senescence-like neutrophils provides a new perspective on SLE pathophysiology and may pave the way for the development of novel therapies.

## Introduction

Systemic lupus erythematosus (SLE) is a representative autoimmune disorder characterized by a complex pathogenesis and the involvement of multiple organ systems [1]. Therefore, investigating the pathogenesis of SLE is of paramount importance, as it offers a theoretical foundation for the development of targeted therapeutics.

Neutrophils are among the earliest cells in human peripheral blood that respond to microbial invasion [2]. Studies have indicated that neutrophils in patients with lupus present abnormal functions, marked by a diminished capacity for recognizing and clearing apoptotic neutrophils mediated by C1q, calreticulin, and CD91 [3]. Additionally, lupus neutrophils show an enhanced ability to form neutrophil extracellular traps (NETs), which contain substantial amounts of chromatin, double-stranded DNA, and autoantigens, such as granular proteins [4]. Moreover, neutrophils in lupus are subject to ferroptosis, and specific inhibitors of ferroptosis significantly alleviate the severity of the disease in lupus-susceptible mice [5]. These findings imply that neutrophils might play a facilitating role in the pathogenesis of lupus. However, we previously discovered that the elimination of neutrophils at an early stage in a lupus mouse model led to an exacerbation of autoimmunity, further revealing that neutrophils inhibit the IFN-γ response via reactive oxygen species (ROS) production. Both type I interferons and type II interferons (IFNs) are implicated in the pathogenesis of SLE [6]. Neutrophils may play a bidirectional regulatory role in the onset and progression of lupus.

Although neutrophils are typically considered a relatively homogeneous population, emerging evidence indicates the existence of heterogeneity. Under steady-state circumstances, neutrophil heterogeneity might arise from aging and the replenishment of freshly released neutrophils from the bone marrow. As neutrophils age, they gradually lose CD62L expression and upregulate CXCR4 level [7]. These senescence-like neutrophils constitute an overly active subset that enhances the activation of αMβ2 integrin (Mac-1) and increases the formation of NETs under inflammatory conditions. The pro-inflammatory activity of neutrophils is positively correlated with their aging in circulation. The morphology of senescence-like neutrophils is characterized by pronounced nuclear hypersegmentation [8]. However, another subset of mature human neutrophils (CD62L ^dim^CD16 ^bri^) presenting hypersegmented nuclear morphology has been identified as a distinct circulating population of myeloid cells with the capacity to suppress human T-cell proliferation [9].

Given their overlapping and contradictory natures, the functions of senescence-like neutrophils need to be further investigated. Furthermore, the mechanism of senescence-like neutrophils in the pathogenesis of SLE has rarely been reported. This study aimed to explore the role of senescence-like neutrophils in SLE and their contribution to abnormal immunity in patients with lupus.

## Materials and Methods

### Patients

A total of 23 SLE patients were recruited for the study from the Rheumatology and Immunology Department at Shanghai East Hospital between September 2021 and March 2024. All patients were diagnosed in accordance with the 2019 European League Against Rheumatism (EULAR)/American College of Rheumatology (ACR) classification criteria [10]. Additionally, 17 age- and sex-matched individuals without any history of autoimmune diseases or other chronic diseases were included as healthy controls (HCs) from the physical examination center. This research was approved by the Ethics Committee of Shanghai East Hospital (Permit number: 2021-YYS-205). Data related to the age, sex, and serological profiles of the patients were collected ( Table 1).

**Table:** **Table 1** Clinical characteristics of lupus patients

Parameter	SLE ( *n* = 23)	HCs ( *n* = 17)
Age	38.56 ± 4.11	36.70 ± 5.26
SLEDAI	7.96 ± 1.30	/
dsDNA-IgG (IU/mL)	107.94 ± 36.54	/
ESR (mm/h)	36.18 ± 6.32	/
CRP (mg/L)	10.23 ± 2.28	/
C3 (g/L)	0.75 ± 0.06	/
C4 (g/L)	0.13 ± 0.027	/
IgG (g/L)	15.36 ± 1.34	/
IgA (g/L)	2.93 ± 0.34	/
IgM (g/L)	0.84 ± 0.11	/
WBC (×10 ^9^/L)	5.86 ± 0.67	/
Hb (g/L)	105.96 ± 3.72	/
PLT (×10 ^9^/L)	213.91 ± 14.92	/

### Mice

Eight-week-old female C57BL/6J mice, along with female MRL/Mpj and MRL/lpr mice, were included. MRL/MpJ-Faslpr/J (MRL/lpr) mice were procured from Shanghai Slack Laboratory (Shanghai, China) and maintained under specific-pathogen-free conditions at the animal care facility of the Shanghai Institute of Nutrition and Health.

### Antibodies

The monoclonal antibodies utilized for flow cytometry were as follows. The anti-human CXCR4 Brilliant Violet 421 ^TM^ (306581), anti-human CD62L PerCP-Cy5.5 (304823), anti-human CD16 APC (302012), anti-human CD66b PE (392904), anti-human CD3 FITC (300306), anti-human CD3 PE (317308), anti-human CD56 APC (318310), anti-human CD335 (NKp46) BV510 (331924), anti-mouse CXCR4 PE (146506), Annexin V PE (640908), anti-mouse NK1.1 PE (108707), and anti-mouse IFNγ FITC (505806) antibodies were purchased from Biolegend (San Diego, USA). Anti-human TNF-α FITC (554512) was purchased from BD Bioscience (Franklin Lakes, USA). Anti-human IFN-γ FITC (12-7319-42), anti-mouse CD11b APC-cy7 (47-0112-82), anti-mouse CD4 Pacific Blue (48-0042-82), anti-mouse CD45 FITC (11-0451-85), anti-mouse TNF-α PE (12-7321-82), anti-mouse CD3 APC (17-0031-83), anti-mouse CD62L APC (17-0621-83), and MitoSOX ^TM^ Red (M36008) were purchased from Thermo Fisher Scientific (Waltham, USA). Fluorescein di(β-D-galactopyranoside) (FDG) was purchased from MedChemExpress (Monmouth Junction, USA).

### Flow cytometry analysis

Erythrocytes were lysed in an isotonic ice-cold NH _4_Cl solution, followed by centrifugation at 4°C. After lysis, total leukocytes were stained with antibodies for 30 min at 4°C in staining buffer. The cells were washed prior to analysis using a CytoFLEX flow cytometer (Beckman Coulter, Pasadena, USA). Senescence-like neutrophils were defined as CD62L ^lo^CXCR4 ^hi^ cells within the neutrophil population, whereas non-senescent neutrophils were gated as CD62L ^hi^CXCR4 ^lo^ cells.

For intracellular cytokine staining, co-cultured cells were subsequently stained for surface markers, fixed and permeabilized with BD fixation/permeabilization buffer before being stained for intracellular cytokines (IFN-γ or TNF-α).

### Cell preparation

Peripheral blood mononuclear cells (PBMCs) were isolated by centrifugation with a Ficoll-Paque PLUS gradient (GE Healthcare, Wisconsin, USA). The neutrophils were subsequently isolated through dextran sedimentation and hypotonic lysis, as previously described [11]. Two populations of neutrophils are sorted by flow cytometry (Arial II): CD62L ^lo^CXCR4 ^hi^ and CD62L ^hi^CXCR4 ^lo^. Human NK cells or CD4 ^+^ T cells from freshly isolated PBMCs were negatively selected using the MACS NK Cell Isolation kit (130-092-657; Miltenyi Biotec, Bergisch Gladbach, Germany) or the MojoSort™ human CD4 T-Cell Isolation kit (480010; Biolegend). Mouse NK cells or CD4 ^+^ T cells from freshly isolated splenocytes were purified using the MACS NK Cell Isolation kit (130-115-818; Miltenyi Biotec) or the MojoSort™ Mouse CD4 T-Cell Isolation kit (480033; Biolegend). The detailed steps were performed according to the manufacturer’s instructions.

### Cell co-culture

NK cells and CD4 ^+^ T cells were co-cultured with neutrophils at a 1:1 cell ratio. Additionally, catalase (1 μg/mL) was added to the neutrophil culture mixture. For the preparation of the NK cell-neutrophil coculture medium, the following constituents were incorporated: rhIL-21 (20 ng/mL), rhIL-18 (100 ng/mL), fMLP (10 μM), PMA (50 ng/mL), ionomycin (1 μg/mL), and GolgiPlug (10 ng/mL). For the CD4 ^+^ T-cell-neutrophil coculture medium, the components consisting of fMLP (10 μM), PMA (50 ng/mL), ionomycin (1 μg/mL), and GolgiPlug (10 ng/mL) were added to 96-well plates pre-coated with anti-CD3 and anti-CD28 antibodies. The samples were cultivated in a cell incubator at 37°C for 4 h prior to collection for subsequent experiments.

### Statistical analysis

All the data are presented as the mean ± SEM unless otherwise stated. Statistical analysis was conducted using GraphPad Prism software (version 10.1.2). The Mann-Whitney U test was applied to datasets that did not conform to a normal distribution, whereas the *t* test was employed for normally distributed paired samples. Spearman’s rank correlation was adopted to analyze the relationships between two continuous variables that were not normally distributed. Statistical significance was defined when *P*  < 0.05.

## Results

### Neutrophil senescence is augmented in SLE patients and correlated with SLE disease activity

**Figure 1:**
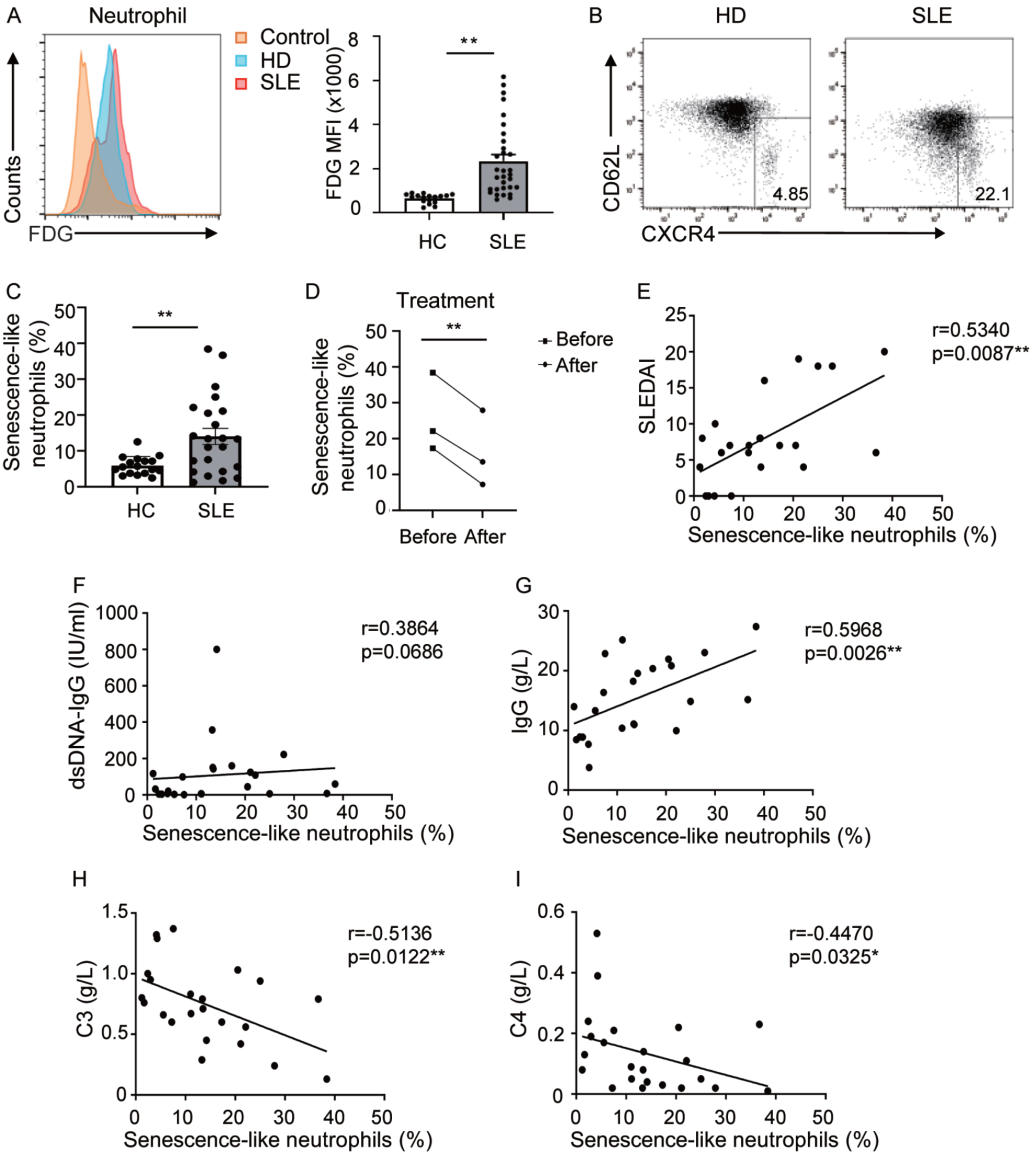
Figure 1 The percentage of senescence-like neutrophils is elevated in SLE patients and is correlated with clinical parameters (A) Flow cytometric analysis of the MFI of FDG in neutrophils between SLE patients and HCs. Data are presented as representative plots (left) and summary graphs (right). (B) Flow cytometric analysis of the proportion of senescence-like neutrophils (CD62LloCXCR4hi) between SLE patients and HCs. (C) Scatter plots depicting the percentages of senescence-like neutrophils. (D) Comparison of the proportion of senescence-like neutrophils following treatment. These patients received combined treatment with glucocorticoids and immunosuppressants (such as mycophenolate mofetil). Correlation between the frequency of senescence-like neutrophils from lupus patients and the SLEDAI (E) and the serum levels of anti-dsDNA-IgG (F), IgG (G), C3 (H), and C4 (I). Data are presented as the mean ± SEM from at least three independent experiments. FDG, fluorescein di(β-D-galactopyranoside); HC, healthy control; SLE, systemic lupus erythematosus; MFI, mean fluorescence intensity; SLEDAI, SLE disease activity index; C3, complement 3; C4, complement 4.

To explore the pronounced senescence of peripheral blood neutrophils in patients with SLE, we initially evaluated the cellular senescence of neutrophils with FDG [12]. Our findings demonstrated that neutrophils from SLE patients displayed a propensity for senescence ( Figure 1A). We subsequently compared the proportion of senescence-like neutrophils (CXCR4 ^hi^CD62L ^lo^) in the peripheral blood of SLE patients with that in the peripheral blood of HCs. The data revealed a significant increase in the percentage of senescence-like neutrophils among SLE patients ( *P*  < 0.05), accounting for approximately 15% of all neutrophils, in contrast to only approximately 5% in HCs ( Figure 1B,C). Moreover, we discovered that the proportion of senescence-like neutrophils decreased markedly following treatment ( Figure 1D).

To further illuminate these observations, we analyzed the correlations between senescence-like neutrophils and lupus-related clinical parameters. The frequency of senescence-like neutrophils was positively associated with the SLE disease activity index (SLEDAI) and immunoglobulin G (IgG) levels ( Figure 1E,G). Conversely, a negative correlation was detected between serum complement levels and the percentage of senescence-like neutrophils ( Figure 1H,I). Although not statistically significant, a modest trend was observed between senescence-like neutrophils and serum anti-dsDNA antibody levels ( Figure 1F). Based on the average proportion of senescence-like neutrophils in patients with SLE, these patients were classified into a low proportion group (< 14%) and a high proportion group (> 14%). Compared with the lower proportion group, the higher proportion group presented greater disease activity ( Table 2). Additionally, correlations were observed between the levels of ESR, C3, IgG, and IgM; and the proportion of senescence-like neutrophils ( Table 2). Although there was no significant difference in anti-dsDNA levels between the two groups, the average level of anti-dsDNA was significantly elevated in the high proportion group ( Table 2). Further studies with larger sample sizes are needed to validate this correlation. Collectively, these results indicate that senescence-like neutrophils are expanded in SLE patients and might serve as indicators of lupus disease activity.

**Table:** **Table 2** Correlation between clinical characteristics of SLE patients and the proportion of senescence-like neutrophils

Parameter	Low ( *n* = 14)	High ( *n* = 9)	*P* value
Senescence-like neutrophils (%)	7.096 ± 1.224	24.800 ± 2.749	< 0.0001****
SLEDAI	4.857 ± 0.948	12.778 ± 2.191	0.0011***
dsDNA-IgG (IU/mL)	67.801 ± 26.869	170.373 ± 82.204	0.1762
ESR (mm/h)	24.656 ± 6.205	54.111 ± 10.845	0.0191*
CRP (mg/L)	8.466 ± 2.341	12.982 ± 4.603	0.3458
C3 (g/L))	0.860 ± 0.081	0.573 ± 0.101	0.0382*
C4 (g/L))	0.167 ± 0.038	0.081 ± 0.029	0.1228
IgG (g/L)	12.864 ± 1.601	19.233 ± 1.723	0.016*
IgA (g/L)	2.521 ± 0.463	3.520 ± 0.520	0.1721
IgM (g/L)	0.644 ± 0.106	1.112 ± 0.191	0.0318*
WBC (×10 ^9^/L)	6.446 ± 0.657	4.944 ± 1.371	0.2833
Hb (g/L)	109.571 ± 5.095	100.333 ± 5.017	0.234
PLT (×10 ^9^/L)	213.786 ± 20.480	214.111 ± 22.431	0.9918

### Senescence-like neutrophils from SLE patients impair the immunosuppressive effects of NK cells

Although our data suggested an increase in neutrophil aging among lupus patients, the specific functions of senescence-like neutrophils remain unclear. Therefore, we monitored the function of senescence-like neutrophils in comparison with that of non-senescent neutrophils (CD62L ^hi^CXCR4 ^lo^). Our previous research demonstrated that neutrophils suppress IL-15 expression via ROS, thereby indirectly inhibiting NK cell-mediated IFN-γ production [6].

**Figure 2:**
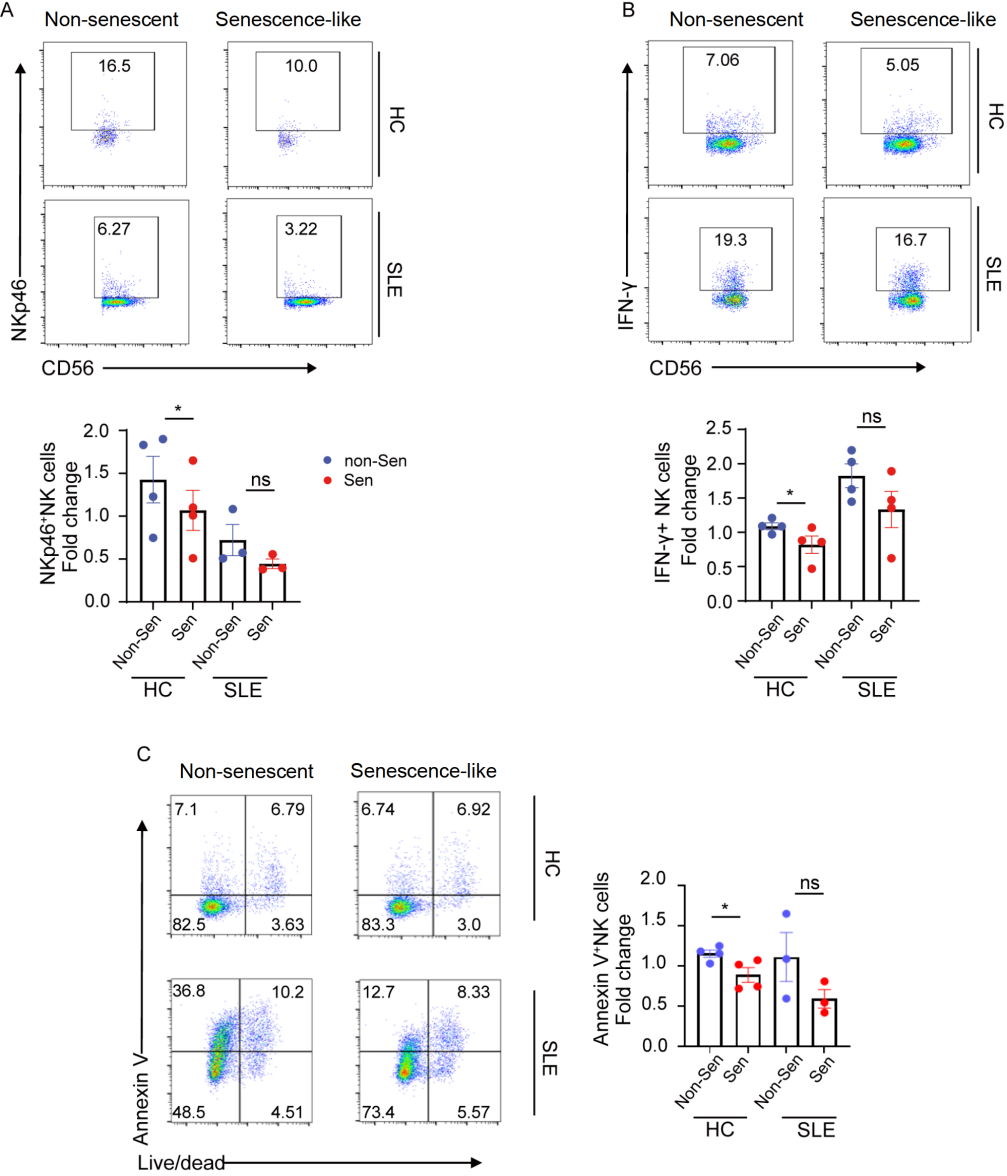
Figure 2 Senescence-like neutrophils from patients with SLE impair the immunosuppressive functions of NK cells NK cells from HCs were co-cultured with non-senescent (CD62LhiCXCR4lo) or senescence-like (CD62LloCXCR4hi) neutrophils from the peripheral blood of HCs or SLE patients. Flow cytometric analysis of the proportions of NKp46+ NK (A) and IFN-γ+ NK cells (B) in the co-culture system. (C) Flow cytometry analysis of the proportion of Annexin V+ NK cells in the co-culture system. Data are presented as representative plots (left) and summary graphs (right). Data are presented as the mean ± SEM from at least three independent experiments. *We elected to present the form as a relative control ratio. This control was chosen based on the expression of the corresponding molecules when the NK cells were cultivated alone in each experiment. Non-Sen: non-senescent neutrophils; Sen: senescence-like neutrophils.

To investigate the direct effects of senescence-like neutrophils on NK cells, we cocultured NK cells from HCs with either senescence-like neutrophils or nonsenescent neutrophils derived from HCs or lupus patients, respectively. NKp46, a surface molecule of NK cells, binds to its ligands, leading to the activation of NK cells and the secretion of IFN-γ [13]. Under co-culture conditions, the expression of NKp46 on NK cells and IFN-γ production were significantly lower when these cells were exposed to senescence-like neutrophils from HCs, suggesting that senescence-like neutrophils exert an inhibitory effect on NK cells under physiological circumstances ( Figure 2A,B). Conversely, senescence-like neutrophils from lupus patients lost this inhibitory ability ( Figure 2A,B). Furthermore, the observed suppressive effect was not attributed to the induction of NK cell apoptosis via neutrophil cytotoxicity ( Figure 2C). Moreover, the non-senescent neutrophil and senescence-like neutrophil subsets from SLE patients presented similar levels of NK cell apoptosis in our co-cultures ( Figure 2C).

### Senescence-like neutrophils from SLE patients exhibit compromised inhibitory effects on CD4 ^+^ T cells

**Figure 3:**
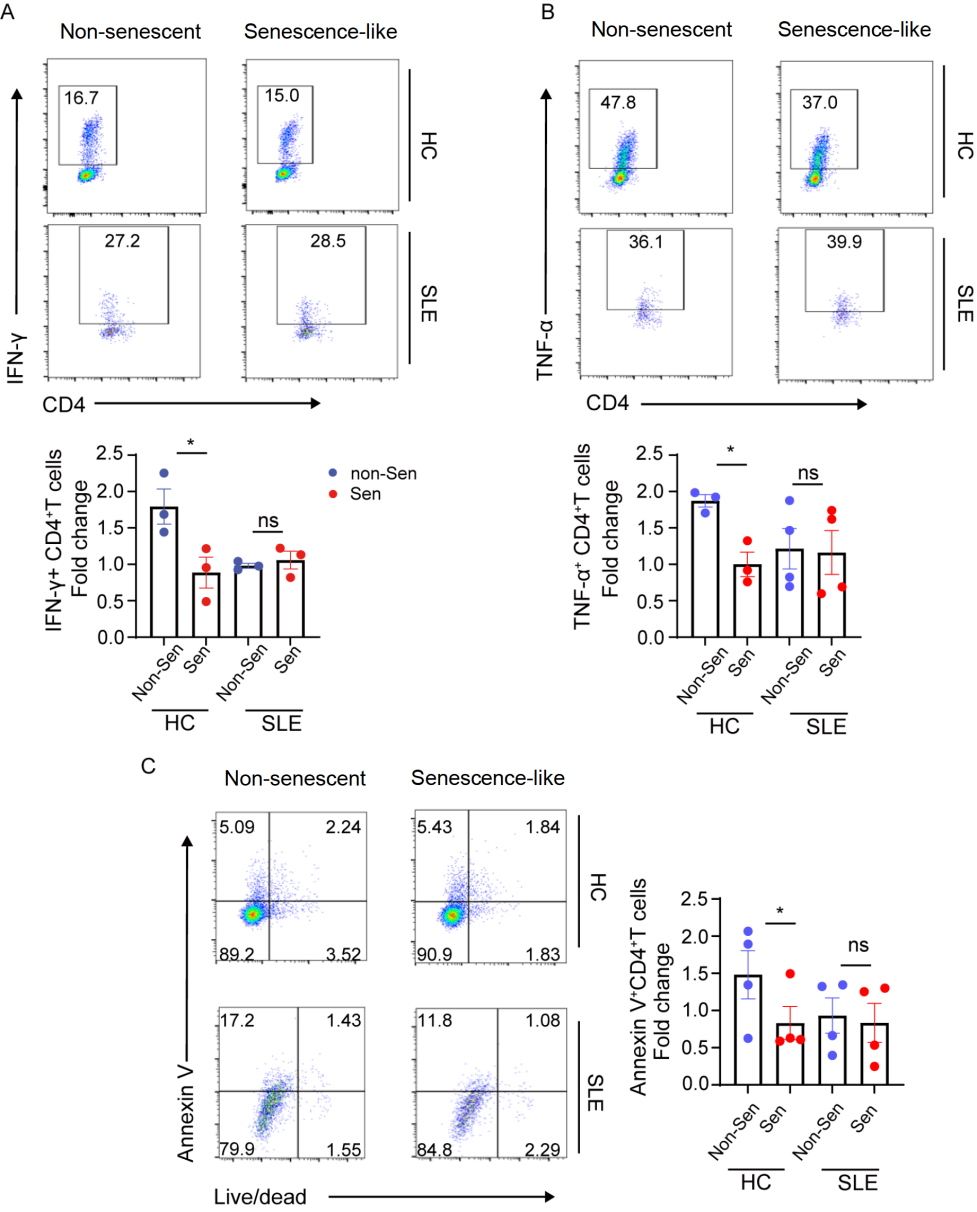
Figure 3 The capacity of senescence-like neutrophils to inhibit CD4 ^+^ T-cell activation is compromised in SLE patients CD4+ T cells from HCs were co-cultured with non-senescent and senescence-like neutrophils from the peripheral blood of HCs or SLE patients. Flow cytometric analysis of the proportions of IFN-γ+CD4+ T cells (A) and TNF-α+CD4+ T cells (B) in the co-culture system. (C) Flow cytometric analysis of the proportion of Annexin V+CD4+ T cells in the co-culture system. Data are presented as representative plots (left) and summary graphs (right). Data are presented as the mean ± SEM from at least three independent experiments. *We elected to present the form as a relative control ratio. This control was chosen based on the expression of the corresponding molecules when CD4+ T cells were cultivated alone in each experiment. Non-Sen: non-senescent neutrophils; Sen: senescence-like neutrophils.

A previous study revealed a distinctive subset of mature human neutrophils that can suppress T-cell proliferation [9]. Therefore, we further explored whether senescence-like neutrophils exert suppressive effects on CD4 ^+^ T cells. We conducted co-culture experiments in which peripheral CD4 ^+^ T cells from HCs were directly incubated with senescence-like or non-senescent neutrophils from lupus patients or HCs. A lower percentage of IFN-γ ^+^CD4 ^+^ and TNF-α ^+^CD4 ^+^ T cells was observed in the group of senescence-like neutrophils than in the group of non-senescent neutrophils from both HCs. Nevertheless, this disparity was not detected when CD4 ^+^ T cells were co-cultured with senescence-like neutrophils from lupus patients ( Figure 3A,B). Similarly, the suppressive effect was not attributed to the induction of CD4 ^+^ T-cell apoptosis via neutrophil cytotoxicity ( Figure 3C). These results indicate that senescence-like neutrophils from lupus patients exhibit impaired suppressive effects on NK and CD4 ^+^ T cells.

### Identification of the impaired suppressive function of senescence-like neutrophils in lupus mice

**Figure 4:**
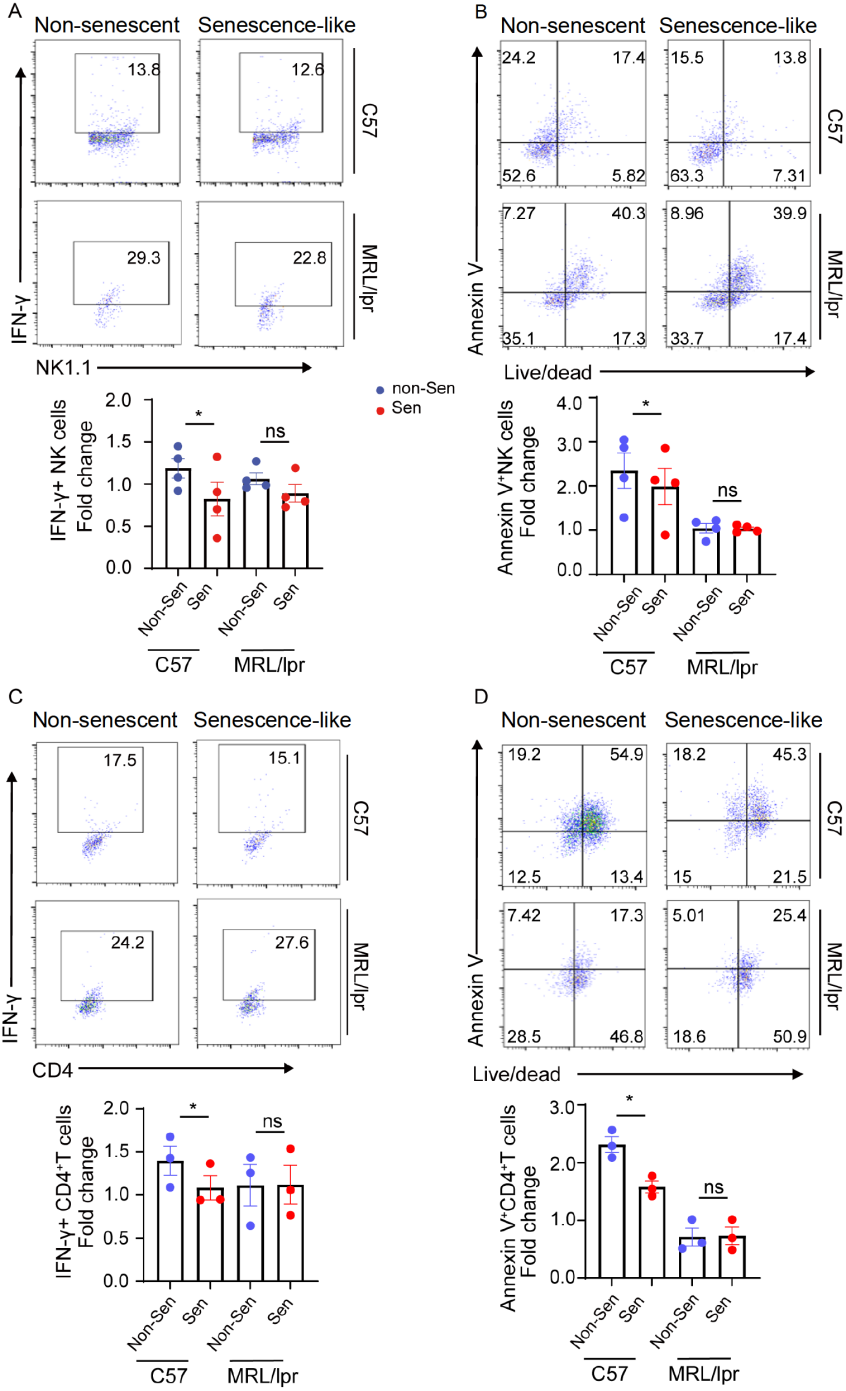
Figure 4 The suppressive function of senescence-like neutrophils is compromised in mouse models of lupus NK or CD4+ T cells from C57BL/6 mice were co-cultured with non-senescent or senescence-like neutrophils from the peripheral blood of C57BL/6 or MRL/lpr mice, respectively. (A) Flow cytometric analysis of the proportion of IFN-γ+ NK cells in the co-culture system. (B) Flow cytometric analysis of the proportion of Annexin V+ NK cells in the co-culture system. (C) Flow cytometric analysis of the proportion of IFN-γ+CD4+ T cells in the co-culture system. (D) Flow cytometric analysis of the proportion of Annexin V+CD4+ T cells in the co-culture system. Data are presented as representative plots (left) and summary graphs (right). Data are presented as the mean ± SEM from at least three independent experiments. *We elected to present the form as a relative control ratio. This control was chosen based on the expression of the corresponding molecules when NK cells or CD4+ T cells were cultivated alone in each experiment. Non-Sen: non-senescent neutrophils; Sen: senescence-like neutrophils.

Given that senescence-like human neutrophils can inhibit the secretion of cytokines from NK cells and CD4 ^+^ T cells, we isolated neutrophils, NK cells, and CD4 ^+^ T cells from wild-type and MRL/lpr lupus mice to validate the inhibitory function of senescence-like neutrophils. Consistent with the findings in humans, we noted similar suppressive functions in senescence-like neutrophils derived from mice ( Figure 4A–D). Furthermore, the suppressive ability of senescence-like neutrophils from lupus mice was diminished ( Figure 4A–D).

### ROS contributes to the immunosuppressive effects of senescence-like neutrophils

**Figure 5:**
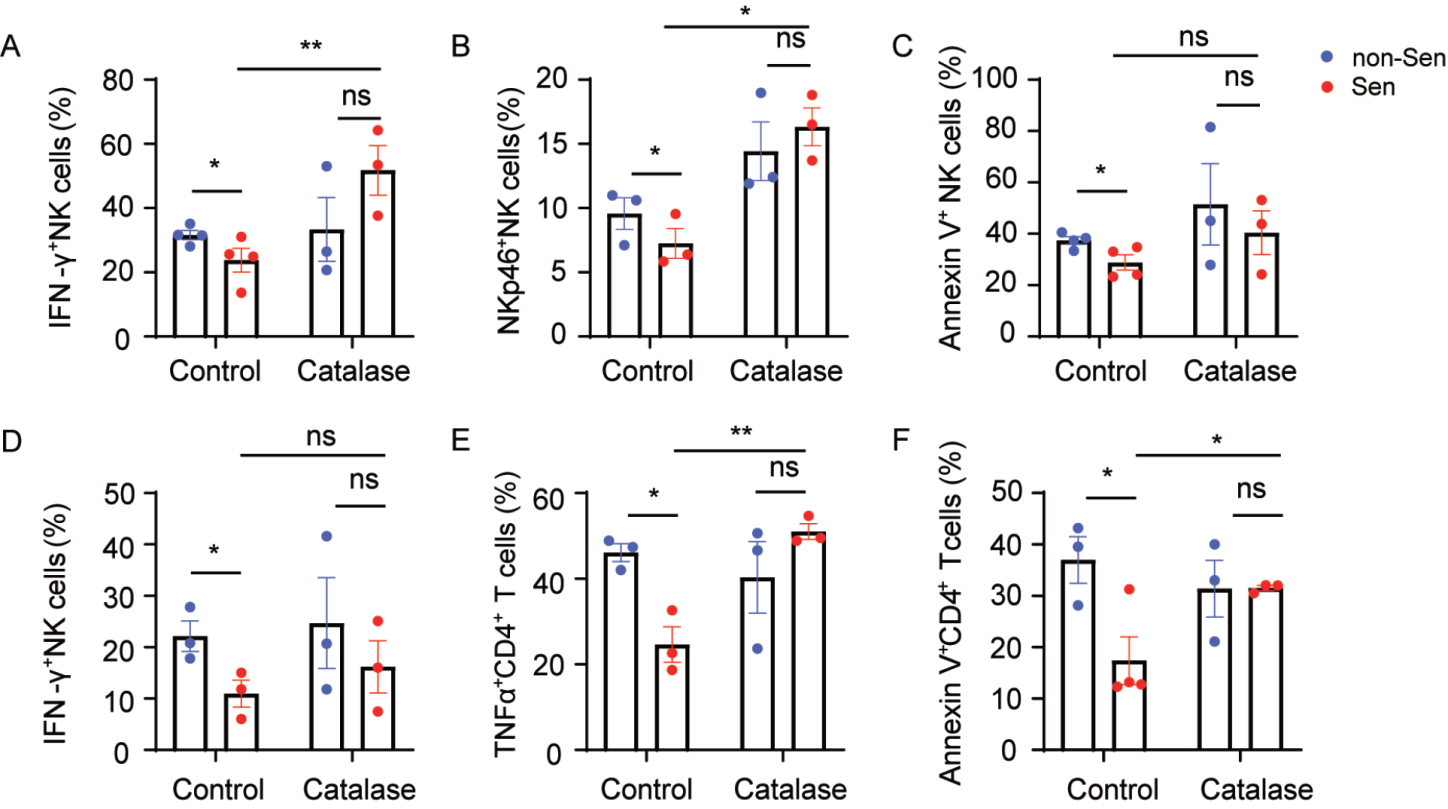
Figure 5 Human senescence-like neutrophils exert immunosuppressive effects via ROS (A–C) NK cells from HCs were co-cultured with non-senescent and senescence-like neutrophils from HCs in the absence or presence of catalase (1 μg/mL). (D–F) CD4+ T cells from HCs were co-cultured with non-senescent and senescence-like neutrophils from the peripheral blood of HCs in the absence or presence of catalase (1 μg/mL). Data are presented as the mean ± SEM from at least three independent experiments. Non-Sen: non-senescent neutrophils; Sen: senescence-like neutrophils.

Neutrophils can release ROS locally at immunological synapses through direct interactions with T cells, thereby influencing T-cell function [9]. Additionally, ROS have been implicated in immune suppression mediated by polymorphonuclear myeloid-derived suppressor cells (PMN-MDSCs) [14]. Consequently, we investigated whether ROS production contributes to the suppressive effects of this neutrophil subset. Indeed, the hydrogen peroxide (H _2_O _2_) scavenger catalase was capable of partially restoring NK and T-cell function under co-culture conditions, mimicking the effects of senescence-like neutrophils derived from SLE patients ( Figure 5A–F). Furthermore, upon the elimination of ROS, a marked increase in the secretion levels of cytokines and surface molecules from both NK cells and CD4 ^+^ T cells was observed when these cells were co-cultured with senescence-like neutrophils ( Figure 5A,B,D,E). This finding implies that senescence-like neutrophils might exert inhibitory functions via ROS. We further verified that senescence-like neutrophils derived from mice also necessitate the presence of ROS ( Supplementary Figure S1A–E). In the lupus mouse model, we discovered that, in both control mice and lupus mice, the capacity of senescent-like neutrophils to generate ROS was higher than that of non-senescent neutrophils. Additionally, the ability of neutrophils derived from lupus mice to produce ROS was significantly lower than that of control mice ( Supplementary Figure S1F). These findings suggest that senescence-like neutrophils might partially exert their immunosuppressive functions through ROS.

## Discussion

SLE is characterized by autoimmunity against nuclear antigens and can involve multiple organs, such as the skin, kidneys, and joints. A crucial pathogenic feature of SLE is the presence of anti-DNA antibodies, which form DNA-containing immune complexes and stimulate the production of IFN-α from plasmacytoid dendritic cells (pDCs). Recent studies have indicated that the extracellular DNA involved in the activation of pDCs originates from neutrophil extracellular traps (NETs) [ 15, 16]. DNA-containing immune complexes activate neutrophils and promote NET formation, thereby increasing pDC activation and IFN-α production through a TLR9-dependent mechanism [ 15, 16]. These findings emphasize the critical role of neutrophil activation and NET formation in the pathogenesis of SLE in humans.

Neutrophils play crucial roles in combating pathogens and are indispensable for tissue repair and immune system regulation [17]. These cells have an extremely brief lifespan in peripheral blood and undergo aging subsequent to their release from the bone marrow [2]. In both chronic and acute inflammatory microenvironments, neutrophils are subjected to prolonged stimulation, which can induce a typical senescence-associated secretory phenotype (SASP). This phenotype leads to the secretion of various pro-inflammatory factors, such as IL-6, IL-8, and other chemokines. The release of these factors not only exacerbates local inflammation but also may trigger systemic inflammatory responses, further worsening pathological conditions.

Senescence-like neutrophils display elevated levels of CXCR4 expression and reduced levels of CD62L, thereby increasing their chemotactic capacity [7]. These senescence-like neutrophils can accumulate in the lymphoid tissues of healthy elderly mice [18]. Compared with non-senescent neutrophils, senescence-like neutrophils demonstrate significantly heightened pro-inflammatory activity [7]. Previous investigations have demonstrated that the microbiota governs the population of senescence-like neutrophils, which in turn influences both acute vaso-occlusive crises and chronic tissue damage in sickle cell disease (SCD) [7]. Furthermore, the participation of senescence-like neutrophils in other conditions, such as interstitial lung disease, HIV, and sepsis, has been progressively elucidated [ 19– 21]. Our observations revealed a marked increase in the frequency of CD62L ^lo^CXCR4 ^hi^ senescence-like neutrophils in SLE patients compared with HCs. Moreover, the percentage of senescence-like neutrophils was correlated with the SLEDAI score and serum IgG and complement levels, suggesting their involvement in the inflammatory response underlying the etiology of SLE.

Previous studies have focused primarily on elucidating the pro-inflammatory functions of senescence-like neutrophils, and no reports are available regarding whether senescence-like neutrophils possess inhibitory functions. To date, it has been established that a certain subset of neutrophils, in addition to PMN-MDSCs, exhibit inhibitory functions. As previously stated, the interplay among pDCs, NK cells, and neutrophils constitutes a finely tuned network, as demonstrated in our earlier study [6]. In this study, we validated, for the first time, the direct inhibitory effect of senescence-like neutrophils on NK and CD4 ^+^ T cells. Additionally, we discovered that the suppressive function of neutrophils was significantly compromised in patients with lupus.

To elucidate the specific mechanisms through which senescence-like neutrophils inhibit the functions of NK and CD4 ^+^ T cells, we observed a notable reduction in the suppressive capacity of senescence-like neutrophils upon the removal of ROS. These findings imply that senescence-like neutrophils might exert their immunosuppressive functions via ROS. Our previous experiments indicated that the levels of ROS in the serum of SLE patients were significantly greater than those in the serum of HCs. ROS can induce oncogene-induced senescence (OIS) [22], suggesting that peripheral neutrophils in SLE may undergo a senescence process due to continuous ROS induction. Intriguingly, despite excessive ROS expression, we discovered that the ability of senescence-like neutrophils derived from lupus mice to produce ROS was significantly lower than that of control mice, thereby resulting in compromised suppressive functions. Indeed, previous studies have shown that senescence-like neutrophils exhibit enhanced functional activation and the ability to form NETs. In our study, we aimed to elucidate the suppressive mechanisms of senescence-like neutrophils. In light of the pro-inflammatory effects associated with NETs, we did not compare NET release between senescence-like neutrophils and non-senescent neutrophils from SLE patients. Given the significance of NETs for the function of neutrophils, we will confirm the release of NETs with senescence-like neutrophils in our future studies.

Overall, for the first time, we present evidence suggesting that dysregulated senescence-like neutrophils might play a crucial role in the pathogenesis of lupus. Our findings could serve as valuable biomarkers for evaluating disease severity. The use of senescence-like neutrophils might fulfil the long-desired goal of monitoring the disease status in patients with SLE. Furthermore, the suppressive function of senescence-like neutrophils is compromised in patients with lupus. We hypothesize that a subpopulation of senescence-like neutrophils possesses immunosuppressive capabilities under physiological circumstances. Nevertheless, in the context of lupus, the suppressive capacity of this subset is undermined, leading to an abnormal pro-inflammatory disease state. This observation, however, demands further investigation for validation. Therefore, the ability to track senescence-like neutrophils may facilitate the development of novel strategies aimed at optimizing and personalizing the diagnosis and treatment of autoantibody-mediated diseases.

Nevertheless, there are several limitations and shortcomings that need to be addressed in the future. First, the sample size of patients in this study was relatively small, thereby mandating an increase in sample size for further validation of our results. Additionally, not all patients were newly diagnosed; some were receiving different lines of treatment. As a consequence, the administration of diverse drugs, along with immunological alterations, might influence the results due to disease progression. Second, our study merely analyzed the total senescence-like neutrophil subset, and further exploration is necessary to determine whether other markers exist for differentiating between pro-inflammatory and suppressive subsets.

## Supporting information

24738supplementary_Figure_1

## Supplementary Data

Supplementary data is available at *Acta Biochimica et Biophysica Sinica* online.

## References

[1] Hoi A, Igel T, Mok CC, Arnaud L (2024). Systemic lupus erythematosus. Lancet.

[2] Ng LG, Ostuni R, Hidalgo A (2019). Heterogeneity of neutrophils. Nat Rev Immunol.

[3] Donnelly S, Roake W, Brown S, Young P, Naik H, Wordsworth P, Isenberg DA (2006). Impaired recognition of apoptotic neutrophils by the C1q/calreticulin and CD91 pathway in systemic lupus erythematosus. Arthritis Rheumatism.

[4] Liu Y, Kaplan MJ (2021). Neutrophil dysregulation in the pathogenesis of systemic lupus erythematosus. Rheumatic Dis Clin N Am.

[5] Li P, Jiang M, Li K, Li H, Zhou Y, Xiao X, Xu Y (2021). Glutathione peroxidase 4–regulated neutrophil ferroptosis induces systemic autoimmunity. Nat Immunol.

[6] Huang X, Li J, Dorta-Estremera S, Di Domizio J, Anthony SM, Watowich SS, Popkin D (2015). Neutrophils regulate humoral autoimmunity by restricting interferon-γ production via the generation of reactive oxygen species. Cell Rep.

[7] Zhang D, Chen G, Manwani D, Mortha A, Xu C, Faith JJ, Burk RD (2015). Neutrophil ageing is regulated by the microbiome. Nature.

[8] Casanova-Acebes M, Pitaval C, Weiss LA, Nombela-Arrieta C, Chèvre R, A-González N, Kunisaki Y (2013). Rhythmic modulation of the hematopoietic niche through neutrophil clearance. Cell.

[9] Pillay J, Kamp VM, van Hoffen E, Visser T, Tak T, Lammers JW, Ulfman LH (2012). A subset of neutrophils in human systemic inflammation inhibits T cell responses through Mac-1. J Clin Invest.

[10] Aringer M, Costenbader K, Daikh D, Brinks R, Mosca M, Ramsey-Goldman R, Smolen JS (2019). 2019 European league against rheumatism/american college of rheumatology classification criteria for systemic lupus erythematosus. Arthritis Rheumatology.

[11] Risnik D, Podaza E, Almejún MB, Colado A, Elías EE, Bezares RF, Fernández-Grecco H (2017). Revisiting the role of interleukin-8 in chronic lymphocytic leukemia. Sci Rep.

[12] Goy E, Martin N, Drullion C, Saas L, Molendi-Coste O, Pineau L, Dombrowicz D (2023). Flow cytometry-based method for efficient sorting of senescent cells. Bio Protoc.

[13] Narni-Mancinelli E, Jaeger BN, Bernat C, Fenis A, Kung S, De Gassart A, Mahmood S (2012). Tuning of natural killer cell reactivity by NKp46 and helios calibrates T cell responses. Science.

[14] Kusmartsev SA, Li Y, Chen SH (2000). Gr-1
^+^ myeloid cells derived from tumor-bearing mice inhibit primary T cell activation induced through CD3/CD28 costimulation. J Immunol.

[15] Lande R, Ganguly D, Facchinetti V, Frasca L, Conrad C, Gregorio J, Meller S (2011). Neutrophils activate plasmacytoid dendritic cells by releasing self-dna-peptide complexes in systemic lupus erythematosus. Sci Transl Med.

[16] Garcia-Romo GS, Caielli S, Vega B, Connolly J, Allantaz F, Xu Z, Punaro M (2011). Netting neutrophils are major inducers of type I IFN production in pediatric systemic lupus erythematosus. Sci Transl Med.

[17] Nicolás-Ávila JÁ, Adrover JM, Hidalgo A (2017). Neutrophils in homeostasis, immunity, and cancer. Immunity.

[18] Tomay F, Wells K, Duong L, Tsu JW, Dye DE, Radley-Crabb HG, Grounds MD (2018). Aged neutrophils accumulate in lymphoid tissues from healthy elderly mice and infiltrate T- and B-cell zones. Immunol Cell Biol.

[19] Kim JH, Podstawka J, Lou Y, Li L, Lee EKS, Divangahi M, Petri B (2018). Aged polymorphonuclear leukocytes cause fibrotic interstitial lung disease in the absence of regulation by B cells. Nat Immunol.

[20] Liu K, Huang HH, Yang T, Jiao YM, Zhang C, Song JW, Zhang JY (2021). Increased neutrophil aging contributes to T cell immune suppression by PD-L1 and arginase-1 in HIV-1 treatment naïve patients. Front Immunol.

[21] Hirano Y, Ode Y, Ochani M, Wang P, Aziz M (2018). Targeting junctional adhesion molecule-C ameliorates sepsis-induced acute lung injury by decreasing CXCR4
^+^ aged neutrophils. J Leukoc Biol.

[22] Lagnado A, Leslie J, Ruchaud-Sparagano M‐, Victorelli S, Hirsova P, Ogrodnik M, Collins AL (2021). Neutrophils induce paracrine telomere dysfunction and senescence in ROS-dependent manner. EMBO J.

