# Supplementary material for: Increased neutrophil senescence is associated with impaired immunosuppressive activity in systemic lupus erythematosus: Neutrophil senescence and immunosuppression in SLE
Source: Acta Biochim Biophys Sin (Shanghai). 2025 Jun 24;57(11):1824–33. doi: 10.3724/abbs.2025047 (PMC12666659; doi:10.3724/abbs.2025047)
Supplement: 24738supplementary_Figure_1 [file 24738supplementary_Figure_1.docx]

**
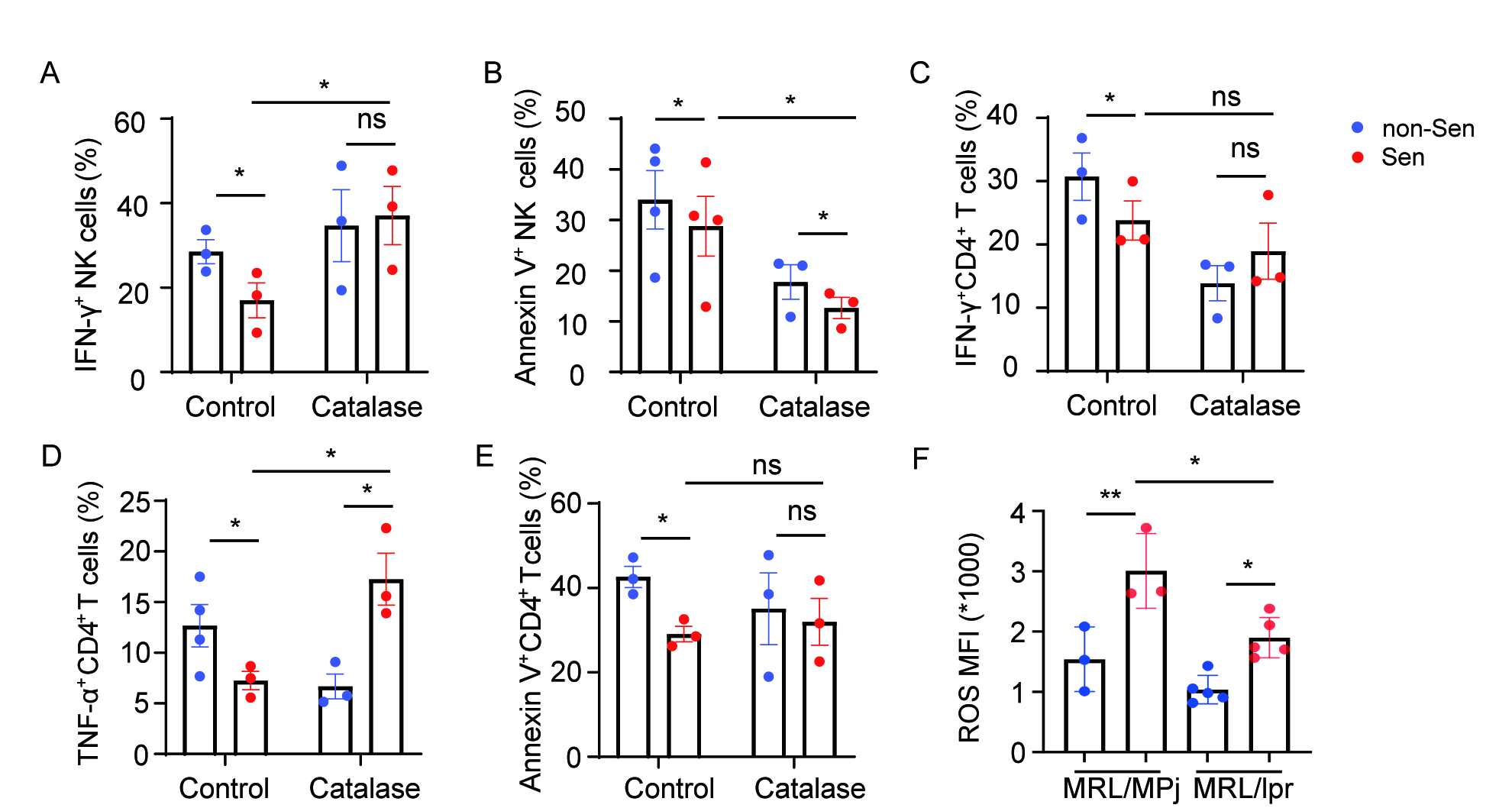
**

**Supplementary Figure S1. Senescence-like neutrophils exert immunosuppressive effects via ROS in mice** (A,B) NK cells from C57BL/6 mice were co-cultured with non-senescent and senescence-like neutrophils from HCs in the absence or presence of catalase (1 μg/mL). (C−E) CD4^+^T cells from HCs were co-cultured with non-senescent and senescence-like neutrophils from peripheral blood of C57BL/6 mice in the absence or presence of catalase (1 μg/mL). (F) Comparison of the expression of ROS from non-senescent and senescence-like neutrophils in both MRL/Mpj control and MRL/lpr lupus mice by MitoSOX^TM^ Red. Data are presented as the mean ± SEM from at least three independent experiments. non-Sen: non-senescent neutrophils; Sen: senescence-like neutrophils.
